# State and Trait Olfactory Markers of Major Depression

**DOI:** 10.1371/journal.pone.0046938

**Published:** 2012-10-03

**Authors:** Marine Naudin, Wissam El-Hage, Marlène Gomes, Philippe Gaillard, Catherine Belzung, Boriana Atanasova

**Affiliations:** 1 INSERM U930 ERL 3106, Université François Rabelais de Tours, Tours, France; 2 Pôle de Psychiatrie, Clinique Psychiatrique Universitaire, CHRU de Tours, Tours, France; University of Pennsylvania School of Medicine, United States of America

## Abstract

Nowadays, depression is a major issue in public health. Because of the partial overlap between the brain structures involved in depression, olfaction and emotion, the study of olfactory function could be a relevant way to find specific cognitive markers of depression. This study aims at determining whether the olfactory impairments are state or trait markers of major depressive episode (MDE) through the study of the olfactory parameters involving the central olfactory pathway. In a pilot study, we evaluated prospectively 18 depressed patients during acute episodes of depression and 6 weeks after antidepressant treatment (escitalopram) against 54 healthy volunteers, matched by age, gender and smoking status. We investigated the participants’ abilities to identify odors (single odors and in binary mixture), to evaluate and discriminate the odors’ intensity, and determine the hedonic valence of odors. The results revealed an “olfactory anhedonia” expressed by decrease of hedonic score for high emotional odorant as potential state marker of MDE. Moreover, these patients experienced an “olfactory negative alliesthesia”, during the odor intensity evaluation, and failed to identify correctly two odorants with opposite valences in a binary iso-mixture, which constitute potential trait markers of the disease. This study provides preliminary evidence for olfactory impairments associated with MDE (state marker) that are persistent after the clinical improvement of depressive symptoms (trait marker). These results could be explained by the chronicity of depression and/or by the impact of therapeutic means used (antidepressant treatment). They need to be confirmed particularly the ones obtained in complex olfactory environment which corresponds a more objective daily life situation.

## Introduction

There is increasing interest in literature to understand the olfactory deficits of depression. An overview of this literature shows conflicting results regarding impairment of all olfactory parameters (i.e., odor threshold, odor identification, discrimination, intensity, familiarity and pleasantness). On the one hand, some studies [1]–[3] showed odor identification deficits in major depressive episode (MDE). Atanasova et al. (2010) [4] demonstrated that olfactory impairments (odor intensity, discrimination and odor pleasantness) depended on the valence of the stimuli. Regarding odor pleasantness, some research teams showed that depressed patients over-evaluated the pleasantness of odors compared to controls [5], [6]. On the other hand, different studies found no significant difference between patients suffering from MDE and healthy controls concerning the odor pleasantness [6]–[9], the odor identification [5], [7], [10]–[14] and the evaluation of odor intensity [5], [6], [9], [15].

The inconsistent findings in this field may be explained by differences in the methodological approaches (e.g., battery of testing, scoring), the clinical type of depression (e.g., seasonal, unipolar, bipolar) and the inclusion criteria of the participants (e.g., medicated or not, types of medications). For instance, the calculation method of the scores of identification, intensity or pleasantness usually considers all the odors, irrespective of the hedonic valence (or pleasantness) of the stimuli. This method does not allow to emphasize the differences between odorants, while it is of particular importance in MDE as anhedonia is a cardinal symptom of the disease (DSM-IV) [16] and the hedonic valence of a component would influence the patient’s ability to identify an odor and evaluate its intensity and pleasantness. This hypothesis is supported by the strong relationships between clinical and sensory anhedonia in the olfactory [9] and the gustatory fields [17]. For these reasons, it is crucial to investigate odor perception using different single odorants in order to evaluate their specific emotional impact on olfactory capabilities. Consequently, the present study used olfactory stimuli with different hedonic valence, and the scores were calculated separately for each odorant.

Furthermore, only one study [4] explored the olfactory abilities in MDE when more complex olfactory stimuli (mixture of odorants) were perceived. Indeed, most of the olfactory studies in mood disorders used single (pure) odorant compounds. This method is incongruent with daily life experiences where a subject experiences more complex olfactory stimuli. Thus, this study proposed an innovative method to investigate odor perception using complex olfactory stimuli. Indeed, we thought that this parameter would be very relevant to the understanding of olfactory impairments in depressed patients in more objective ways. Finally, to our knowledge, few studies have evaluated the effects of the improvement of depressive symptoms on the olfactory abilities, and no study has investigated this aspect in a complex olfactory environment (odorant mixtures). Thus, evaluating the different olfactory parameters during a MDE and after clinical improvement in response to antidepressant treatment will allow us to determine whether the observed olfactory impairments are state- (disappearance of olfactory alterations in clinically improved patients) or trait-related (persistent olfactory alterations after clinical improvement). Indeed, according to Atanasova et al. (2008) [18], olfactory abnormalities might be a cognitive marker for psychiatric conditions, with a specific pattern for each disease.

Thus, the aim of this pilot research was to determine the specific potential olfactory markers for depression by investigating several olfactory parameters during acute depressive phase and when patients were clinically improved. The studied olfactory parameters were the odor identification (identification of single odors and identification of odors in binary iso-intense pleasant/unpleasant mixture), the odor intensity and discrimination evaluation, and the odor hedonic evaluation. We hypothesized that depressed and/or clinically improved patients would have deficits in odor intensity and identification (of single odors), according to the hedonic valence of the stimuli, and that they would have difficulties discriminating different concentrations of pleasant stimuli when compared to controls. Concerning the hedonic evaluations, we hypothesized that depressed and/or clinically improved patients would perceive the pleasant odorants as less pleasant than controls, and the unpleasant odorants as more unpleasant. Lastly, concerning the identification of odors in binary mixture, we hypothesized that depressed and/or clinically improved patients would fail to identify the pleasant odorant compared with unpleasant one.

## Methods

The study was approved by the local ethical committee board (Ethics committee of Tours Ouest-1, France) and conducted in accordance with Good Clinical Practice procedures and the current revision of the Declaration of Helsinki.

### Participants

Eighteen inpatients were recruited consecutively upon admission to the psychiatric ward while seeking treatment for MDE, which lasted more than 15 days. Detailed information of medical history was available in all the cases. Among patients in the depression group, 6 experienced their first episode, 4 their second, and 8 their third episode or more. Each patient was visited by a psychiatrist who made the diagnosis of MDE based on the DSM-IV criteria and using the French version of the Mini International Neuropsychiatric Interview (MINI 5.0.0) [19], [20]. The Montgomery*-*Åsberg Depression Rating Scale (MADRS) [21] was used to assess the severity of depressive symptoms at inclusion (first visit: V1) and after 6 weeks of antidepressant treatment (second visit: V2, 42±2 days after V1). Only patients with a MADRS score ≥28 at V1 were included in the study (mean MADRS score 35.1±4.5).

We excluded patients with DSM-IV psychiatric comorbidity (i.e., psychosis, eating disorder or addiction). The exclusion criteria for all participants comprised also possible brain damage, major medical problems, current substance abuse, allergies, a current cold or a problem with their sense of smell. All subjects were selected on the absence of anosmia to the odorants used in the present study.

After 6 weeks of treatment all patients were clinically improved. Indeed, all of them improved significantly MADRS score (9.1±5.6) and 94% of patients had at least a 50% reduction in baseline MADRS total score. The reduction in the depression score from the first to the second visit (Wilcoxon signed test: V = 171.00, p<0.001) and differences between patients and controls were highly significant (Mann-Witney test; patients V1 and controls: U = 972.00, p<0.001; patients V2 and controls: U = 839.00, p<0.001).

All patients received escitalopram at a flexible dose of 10–20 mg daily, but not necessarily as monotherapy. Indeed, benzodiazepine was administered for insomnia to 6 patients and beta-blocker was prescribed to 2 patients (for hypertension). No other psychotropic agents were used. Drug adherence was monitored and ensured by psychiatric nurses. Patients did not receive specific psychotherapy during their stay at hospital.

Health controls had no personal or family history of any axis I disorder (MINI). They were drug-free and matched to cases on age, gender and smoking status in a ratio of 3∶1. The characteristics of the groups are presented in Table 1.

**Table 1:** Group characteristics.

	Depressed patients (n = 18)	Clinically improved patients (n = 18)	Control subjects (n = 54)
Female/Male ratio	12/6	12/6	36/18
Mean age, years (SD)*	50.1 (13.3)		49.5 (12.5)
Range	20–74		20–74
Somkers/no smokers ration	8/10		24/30
MADRS, mean score (SD)	35.1 (4.5)	9.1 (5.6)	2.33 (2.3)

* Mann-Witney test (U = 474.50; p = 0.89).

### Experimental Procedure

The experimental procedure was clearly explained to all participants. All subjects were informed that they were free to discontinue testing at any time. None of the participants had a reduced capacity/ability to understand the instructions of study and to give her/his consent. The capacity to consent to research of the patients was confirmed by a clinician. All subjects provided a written informed consent prior to testing. They were instructed not to smoke for at least 30–40 min before the study.

### General Design

Prior to the test session, all sensory tasks (evaluation of the odor parameters: pleasantness, familiarity, intensity, and their odor identification) were explained to the participant. Each subject assessed the hedonic aspect, the familiarity and the identification of single odors, before evaluating the odors’ intensity and identification in binary mixture.

Sessions typically lasted for 25 to 30 minutes. The different tests were presented in the same order for all participants. For each task, the presentation order of the different stimuli was balanced across stimuli and was identical for all subjects.

For all experiments, the solutions were made with distilled water (all odorants were soluble in this solvent at the studied concentrations). The odorous solutions were poured into 60 ml brown glass flasks (10 ml per flask). A three-digit random number coded each flask. Earlier experiments [22] showed that each individual optimizes the sniffing parameters to obtain his maximum sensitivity. Therefore, the time allowed for sniffing was not limited, but a minimum 30-second interval between samples was imposed in order to prevent olfactory adaptation.

### Hedonic Aspect, Familiarity and Identification of Single Odors

Firstly, the subjects were invited to smell the eight odorants presented below one after the other. They had to evaluate the pleasantness and the familiarity level of the perceived odors on a 10 cm linear scale labeled at each end (highly unpleasant/highly pleasant; unfamiliar odor/very familiar odor). The resulting response was expressed with a score ranging from 0 to 10. Odor familiarity for all eight odorants was evaluated, in order to investigate a possible influence of this parameter on the olfactory perception. The subject had also to identify the odorant from a list of four descriptors (multiple choice paradigm).

Among the eight studied odorants, 4 were considered as pleasant [Vanillin (6 g/l); 2-phenylethanol, rose (1 ml/l), (E)-cinnamaldehyde, cinnamon (0.25 ml/l) and benzaldehyde, bitter almond (0.5 ml/l)], 2 were neutral [eugenol, clove (0.25 ml/l) and 1-octen-3-ol, mushroom (0.05 ml/l)] and 2 were unpleasant [isovaleric acid, the odor of sweat (0.05 ml/l) and butyric acid, the odor of old cheese (1.6 ml/l)] [23]–[25]. All odorant compounds were supplied by Fisher Scientific Bioblock, Sigma (Illkirch, France). Their concentrations were chosen to be iso-intense.

### Evaluation and Discrimination of Odors’ Intensity

Secondly, subjects had to evaluate the perceived odor intensity of two odorants, one pleasant (2-phenylethanol, PHE) and one unpleasant (isovaleric acid, ISO). These were presented at three different supra-threshold concentration levels: PHE1 = 1 ml/l, PHE2 = 3.5 ml/l PHE3 = 12.5 ml/l, and ISO1 = 0.01 ml/l, ISO2 = 0.05 ml/l ISO3 = 0.25 ml/l. These concentrations were chosen to be iso-intense (PHE1 = ISO1, PHE2 = ISO2, PHE3 = ISO3) and easily differentiated (PHE1≠PHE2≠PHE3, ISO1≠ISO2≠ISO3) in a preliminary test according to the methodology described previously [4]. A 10 cm linear scale labelled at each end (very low intensity/very high intensity) was used to evaluate the perceived odor intensity of all stimuli. When the subjects did not perceive any odor in the flask, they were instructed to not evaluate its intensity.

### Identification of Odors in Binary Mixture

The subjects were asked to identify the perceived odor(s) in a mixture of two odorants presented at iso-intense level, one pleasant (PHE2) and one unpleasant (ISO2). Before the measurement session, the subjects were instructed to smell and to memorize the odor quality of two flasks containing PHE and ISO respectively. The participants were informed that after this, they would have to identify the memorized odors. They knew that the flask may contain one or both odorants at the same time or another stimulus. Thus, subjects had to answer if they thought the sample contained only the 2-phenylethanol (PHE), only isovaleric acid (ISO), both 2-phenylethanol and isovaleric acid (PHE+ISO), or different odor/just the solvent (another odor). This last response was added in order to predict an eventual inhibition phenomenon (no perception of the binary mixture) or to predict the formation of a new odor. However, this response was never chosen by any participant.

### Statistical Analysis

Statistical analyses were carried out with non-parametrical tests, because the Levene tests for the homogeneity of variances revealed unequal variance for the majority of the variables and the normal distribution of the data was not always validated (Kolmogorov-Smirnov test).

The Wilcoxon signed test (paired test) was used for each stimulus to compare the pleasantness response, the familiarity level of odors, their intensity response as well as their identification score (over all odorants; the subjects’ identification scores ranged from 0 to 8) between depressed patients and clinically improved patients. The comparison of these parameters between depressed patients and controls and between clinically improved patients and controls was carried out with Mann-Whitney test (unpaired test).

The Chi-squared test was used to test for differences, between groups, in the proportions of subjects choosing all three responses concerning odor identification in the binary mixture: PHE, ISO and PHE+ISO. In the case, that this test showed the presence of a significant difference between groups for their responses, a chi-squared test for each type of the response was carried out in order to know the type of difference between the three groups. The same tests were used also to analysis the odor identification score per odorant.

In order to compare the hedonic responses of all 8 odorants for each group, the Friedman’s paired test (for both patients groups: 8 odors and 18 subjects; for controls’ group: 8 odors and 54 subjects) with Bonferroni correction (α* = α/k; where, α = 0.05 and k is the number of the comparisons performed) was used. The post-hoc Nemenyi procedure permitted two-by-two comparisons of the hedonic score of the different odorants. The same statistical tests were used for the three groups, to study the discrimination power of the three different intensity levels of 2-phenylethanol and isovaleric acid. For this case, the Friedman’s test was carried out on the 3 intensity levels and the 18 subjects for both patients groups and on the 3 intensity levels and the 54 subjects for controls’ group. The post-hoc Nemenyi procedure permitted two-by-two comparisons of the different intensity levels. All statistical analyses were performed at α = 5%. They were conducted using XLSTAT®-Pro, release 5.2.

## Results

### Hedonic Aspect, Familiarity and Identification of Single Odors

The three groups of subjects were able to discriminate the studied odorants according to their hedonic valence (depressed patients: Q = 43.23, p<0.001; clinically improved patients: Q = 63.27, p<0.001 and controls: Q = 237.22, p<0.001). Thus, controls classified the 8 odorants in 3 clusters; the depressives formed 2 clusters, while the clinically improved patients classified odorants according to their hedonic valence in 4 clusters (Table 2).

**Table 2:** Hedonic classification of odors by three groups.

DP				CIP						HC				
Odorant	Ranks	Groups		Odorant	Ranks	Groups				Odorant	Ranks	Groups		
Isovaleric acid	2.6	A		Isovaleric acid	1.8	A				Isovaleric acid	1.7	A		
Butyric acid	2.6	A		Butyric acid	3.1	A	B			Butyric acid	2.5	A	B	
1-Octen-3-ol	3.9	A	B	1-Octen-3-ol	3.4	A	B			1-Octen-3-ol	3.3		B	
Eugenol	4.1	A	B	Eugenol	4.1	A	B	C		Eugenol	3.5		B	
(E)-Cinnamaldehyde	5.4		B	(E)-Cinnamaldehyde	4.8		B	C	D	(E)-Cinnamaldehyde	5.8			C
Vanillin	5.4		B	2-Phenylethanol	6.1			C	D	Benzaldehyde	6.0			C
Benzaldehyde	5.7		B	Vanillin	6.1			C	D	2-Phenylethanol	6.4			C
2-Phenylethanol	6.3		B	Benzaldehyde	6.7				D	Vanillin	6.7			C

Mean ranks of each odorant and odorants ranking obtained by depressed patients [DP] (n = 18), clinically improved patients [CIP] (n = 18) and healthy controls [HC] (n = 54). For each group of the subjects, values with the same letter are not significantly different at α = 5% according to Nemenyi procedure.

Regarding the pleasant odorants, only one compound (benzaldehyde) was perceived as significantly less pleasant by depressed compared to clinically improved patients. This odorant was found as less pleasant by patients than by controls, only during the depressive episode. At 6 weeks, no significant difference remained between groups. Vanillin and (E)-cinnamaldehyde were evaluated as significantly less pleasant by depressed patients at V1 and V2, compared to healthy controls. The hedonic score of 2-phenylethanol was significantly lower for depressed patients at V1 compared to controls (Table 3A).

**Table 3:** Hedonic and familiarity responses of odors by three groups.

A. Odor hedonic response									
Odorant	DP	CIP	p^1^	DP	HC	p^1^	CIP	HC	p^2^
Vanillin	4.9 (2.9)	5.3 (2.4)	0.5	4.9 (2.9)	7.8 (1.8)	<0.001	5.3 (2.4)	7.8 (1.8)	<0.001
2-Phenylethanol	6.2 (2.5)	6.5 (3.1)	0.4	6.2 (2.5)	7.7 (1.9)	0.03	6.5 (3.1)	7.7 (1.9)	0.3
(E)-Cinnamaldehyde	4.2 (3.5)	4.4 (3.0)	1.0	4.2 (3.5)	7.1 (2.4)	0.005	4.4 (3.0)	7.1 (2.4)	0.0006
Benzaldehyde	4.8 (2.5)	6.5 (1.8)	0.01	4.8 (2.5)	7.1 (2.3)	0.0006	6.5 (1.8)	7.1 (2.3)	0.1
Eugenol	2.9 (2.8)	3.5 (3.0)	0.4	2.9 (2.8)	3.6 (2.3)	0.1	3.5 (3.0)	3.6 (2.3)	0.6
1-Octen-3-ol	2.1 (2.1)	2.3 (2.2)	0.5	2.1 (2.1)	3.2 (2.4)	0.051	2.3 (2.2)	3.2 (2.4)	0.09
Isovaleric acid	1.3 (1.7)	0.8 (0.8)	0.9	1.3 (1.7)	1.2 (1.2)	0.8	0.8 (0.8)	1.2 (1.2)	0.6
Butyric acid	1.1 (1.3)	1.9 (2.4)	0.2	1.1 (1.3)	2.4 (1.7)	0.003	1.9 (2.4)	2.4 (1.7)	0.08
**B. Odor familiarity response**									
**Odorant**	**DP**	**CIP**	**p** 1	**DP**	**HC**	**p** 1	**CIP**	**HC**	**P** 2
Vanillin	5.6 (3.4)	5.4 (2.7)	0.9	5.6 (3.4)	7.9 (1.9)	0.02	5.4 (2.7)	7.9 (1.9)	0.0002
2-Phenylethanol	5.1 (2.7)	4.9 (3.3)	0.9	5.1 (2.7)	6.2 (2.6)	0.1	4.9 (3.3)	6.2 (2.6)	0.1
(E)-Cinnamaldehyde	3.9 (3.5)	4.7 (3.0)	0.4	3.9 (3.5)	5.4 (2.7)	0.08	4.7 (3.0)	5.4 (2.7)	0.4
Benzaldehyde	6.7 (2.7)	6.8 (2.6)	0.8	6.7 (2.7)	7.0 (2.3)	0.7	6.8 (2.6)	7.0 (2.3)	0.8
Eugenol	5.2 (3.3)	5.9 (3.0)	0.5	5.2 (3.3)	5.8 (3.0)	0.6	5.9 (3.0)	5.8 (3.0)	0.9
1-Octen-3-ol	3.5 (3.3)	3.9 (3.0)	0.2	3.5 (3.3)	5.0 (2.8)	0.06	3.9 (3.0)	5.0 (2.8)	0.1
Isovaleric acid	2.0 (2.1)	2.2 (3.2)	0.8	2.0 (2.1)	2.5 (2.6)	0.7	2.2 (3.2)	2.5 (2.6)	0.4
Butyric acid	2.2 (2.5)	2.7 (3.1)	0.6	2.2 (2.5)	2.7 (2.7)	0.6	2.7 (3.1)	2.7 (2.7)	0.9

1 Wilcoxon signed test;

2 Mann-Withney test.

Mean values (SD) of hedonic (A) and familiarity (B) responses of eight odorants obtained by the three groups of subjects: depressed patients [DP] (n = 18), clinically improved patients [CIP] (n = 18) and healthy controls [HC] (n = 54).

Concerning the unpleasant odorants, only butyric acid was perceived as significantly more unpleasant by depressed subjects than controls.

Regarding the neutral odorants, no significant difference was found between the three groups for 1-octen-3-ol and eugenol (Tables 3A).

There was no significant difference between the groups concerning their evaluation of the familiarity of all odorants (for each odorant p>0.05), except for vanillin. Vanillin was evaluated as less familiar by depressed and clinically improved patients compared to controls (Tables 3B).

Regarding the subjects’ odor identification performances, there was no significant difference between the three groups, considering all odorants (K = 1.60, p = 0.45) or each odorant independently (χ^2^ = 2.57, p = 1.0).

### Evaluation and Discrimination Concerning the Intensity of Odors

**Figure 1:**
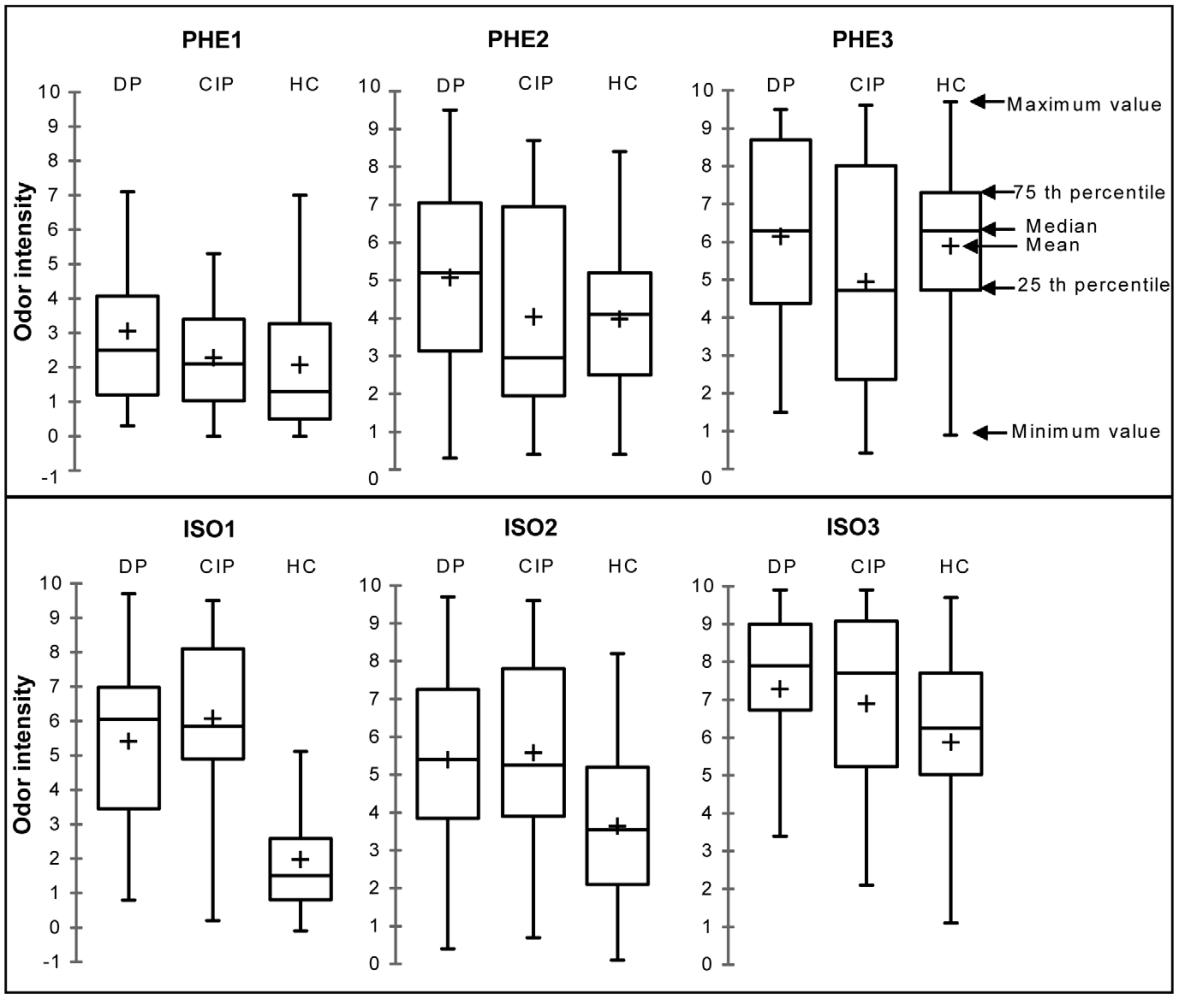
Odor intensity evaluation. Between-groups comparison of odor intensity scores of the three concentration levels of 2-phenylethanol (PHE) and isovaleric acid (ISO) evaluated in depressed patients [DP] (n = 18), in clinically improved patients [CIP] (n = 18) and in healthy controls [HC] (n = 54).

There was no significant difference between the three groups concerning the evaluation of the intensity of the three concentration levels of the pleasant odorant: PHE1 [V1 versus V2 (V = 102.50, p = 0.46); V1 versus controls (U = 605.50, p = 0.12); V2 versus controls (U = 551.00, p = 0.40)], PHE2 [V1 versus V2 (V = 115.50, p = 0.19); V1 versus controls (U = 605.50, p = 0.12); V2 versus controls (U = 471.00, p = 0.85)] and PHE3 [V1 versus V2 (V = 123.50, p = 0.10); V1 versus controls (U = 508.50, p = 0.77); V2 versus controls (U = 406.00, p = 0.30)] (Figure 1).

Evaluating the unpleasant odorant, two concentrations were perceived as significantly more intense by depressed subjects at V1 and V2, compared to controls: ISO1 [V1 versus controls (U = 832.00, p<0.001); V2 versus controls (U = 868.00, p<0.001)] and ISO2 [V1 versus controls (U = 676.00, p = 0.01); V2 versus controls (U = 688.50, p = 0.008)]. After 6 weeks treatments, clinically improved patients were comparable to controls in their perception of the odor intensity at the highest concentration ISO3 (U = 616.00, p = 0.09). There was no significant difference between depressed patients and clinically improved patients at any concentration level of isovaleric acid (p>0.05) (Figure 1).

Concerning the discrimination of odor intensity (Table 4), we found that for both pleasant (PHE) and unpleasant (ISO) odorants, patients were unable to discriminate correctly the three different concentration levels during the MDE (PHE: Q = 14.74, p = 0.001; ISO: Q = 6.85, p = 0.03) and after 6 weeks of antidepressant treatment (PHE: Q = 11.41, p = 0.003; ISO: Q = 2.94, p = 2.23), whereas controls succeeded in this discrimination task (PHE: Q = 58.80, p<0.001; ISO: Q = 59.70, p<0.001).

**Table 4:** Discrimination of odor intensity by three groups.

	2-phenylethanol (PHE)			Isovaleric acid (ISO)		
Concentration level	DP	CIP	HC	DP	CIP	HC
**C1**	3.1 (2.4)^A^	2.3 (1.6)^A^	2.1 (1.9)^A^	5.4 (2.6)^A^	6.1 (2.4)^A^	2.1 (1.7)^A^
**C2**	5.1 (2.7)^B^	4.0 (2.9)^AB^	4.0 (2.0)^B^	5.4 (2.7)^A^	5.6 (2.8)^A^	3.6 (2.0)^B^
**C3**	6.1 (2.6)^B^	4.9 (3.1)^B^	5.9 (2.5)^C^	7.3 (2.6)^A^	6.9 (2.6)^A^	5.9 (2.4)^C^

Odor intensity mean scores (SD) of 2-phenylethanol (PHE) and isovaleric acid (ISO) evaluated in depressed patients at V1 [DP] (n = 18), in clinically improved patients at V2 [CIP] (n = 18) and in healthy controls [HC] (n = 54). The results must be read in columns: for each odorant, mean values with the same letter are not significantly different at α = 5%, using the Nemenyi procedure.

### Identification of Odors in Binary Mixture

**Figure 2:**
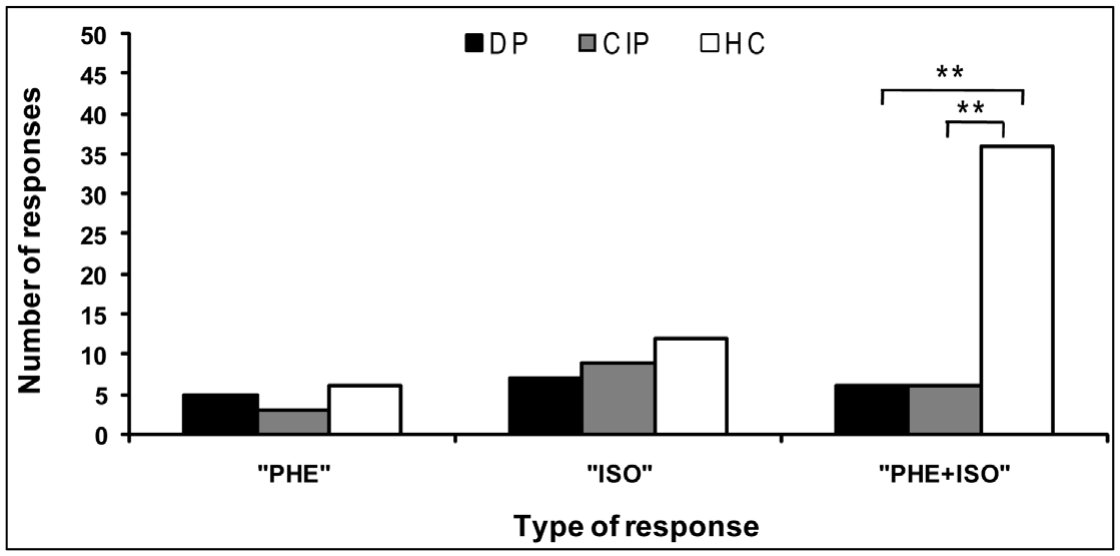
Odors’ identification in binary mixture. Between-groups comparison of the number of responses of three type of responses (PHE: 2-phenylethanol, ISO: isovaleric acid, and PHE+ISO) in depressed patients [DP] (n = 18), in clinically improved patients [CIP] (n = 18) and in healthy controls (HC, n = 54). **: p≤0.01 (Chi-squared test).

The results showed the presence of significant difference between groups, in the proportions of subjects choosing all three responses (χ^2^ = 10.71, p = 0.03). Only 33% of depressed and clinically improved patients were able to identify both odorants simultaneously in the binary mixture (PHE+ISO), while 67% of controls recognized the binary mixture (significant difference between patients and controls: χ^2^ = 9.6, p = 0.008). For the two others responses, no significant difference was found between the three groups for PHE (χ^2^ = 2.9, p = 0.24) or ISO (χ^2^ = 5.50, p = 0.06) (Figure 2).

## Discussion

In the present study, we assessed the olfactory performances during a MDE using 8 single odors with different hedonic valence and two odors with opposite valence in binary mixture. Thus, the study aims at giving preliminary results concerning the state and trait olfactory alterations associated with a MDE by evaluating the olfactory performances during the acute episode and after clinical improvement (at 6 weeks of antidepressant treatment).

In accordance with the literature [5], [7], [10]–[14], this pilot study confirmed that the olfactory identification abilities were not altered in depressed subjects and did not depend on the hedonic valence of the odorant. The results of other olfactory parameters had put light on some olfactory alterations that could constitute state markers for MDE.

Firstly, the results of the hedonic responses to all 8 odorants showed that healthy controls perceived these odors like pleasant, unpleasant and neutral as already demonstrated in the literature [23]–[25]. However, depressed patients classified the odors in only two clusters, pleasant or unpleasant. At 6 weeks of antidepressant treatment, we observed different clustering of odors, suggesting that the patients’ odor hedonic perception tended to normalize following improvements in depressed mood. These results suggest the presence of the olfactory hedonic evaluation impairment in depressives which could be considered as sate and/or trait markers of MDE. In order to confirm this hypothesis, the hedonic responses for each odorant were compared between the three groups of subjects.

Regarding the hedonic evaluation of the different odors, our results confirm that depressed patients perceived pleasant odorants as less pleasant than controls, but only for the almond odor (benzaldehyde). This result raised the question of why this olfactory bias concerned one odor precisely. In fact, the majority of the participants pointed out that benzaldehyde was a very pleasant odor recalling them the smell of the glue they used at school. So, benzaldehyde was a highly emotional odorant for most of the participants.

This hedonic olfactory bias vanished after 6 weeks of antidepressant treatment. This is the first study to show the concurrent positive effects of escitalopram improving depression and “olfactory anhedonia” (for one highly emotional odorant). Consequently, we can assume that escitalopram restored the olfactory anhedonia bias for benzaldehyde, an odorant with high emotional impact. Indeed, antidepressant treatment is known to improve mood impairments due to an abnormal activation of the amygdala and the orbitofrontal cortex [26]–[30]. These brain structures are also involved in both olfactory and emotional processing [31], [32].

Few previous studies have found opposite results [5], [6], with depressed patients over-evaluating the pleasantness of odors compared to controls. Indeed, Pause et al. (2001) [6] reported that MDE patients rated the odorant citral as more pleasant than did healthy controls. Likewise, Loubion-Pouthier et al. (2006) [5] found that MDE patients over-evaluated the pleasantness of the odorants compared to controls (score calculated as a mean of all odorants). This discrepancy is likely to be understood if we control the emotional value of the tested odorants. This hypothesis needs validation, e.g. by measuring physiological parameters (heart rate, skin conductance, respiratory frequency) that reflect the subject’s emotional reactivity.

Secondly, our preliminary results showed some olfactory alterations that could constitute trait markers for MDE.

Concerning the hedonic valence, two pleasant odorants were evaluated as less pleasant by depressed subjects before and 6 weeks of antidepressant treatment: vanillin and (E)-cinnamaldehyde (cinnamon odor). This result is in accordance with persistent olfactory anhedonia for everyday life odorants (vanillin, cinnamon).

Regarding the odor intensity, our results partly confirm that depressed subjects evaluated the unpleasant stimuli as more intense. Indeed, two concentration levels for unpleasant component were evaluated as significantly more intense by patients even after 6 weeks of antidepressant treatment. We replicate here previous findings [4], confirming the “olfactory negative alliesthesia” in depressed subjects at the quantitative level. In our sample, patients and controls were comparable when evaluating the odor intensity of pleasant stimuli, which was not observed previously [4]. This difference may be explained by the difference in the type of used odorants, their intensity level and their emotional impact on the subjects. Moreover, our depressed group failed to discriminate correctly the three different concentration levels, both for pleasant (2-phenylethanol) and unpleasant stimuli (isovaleric acid). Likewise, this parameter did not improve after the treatment.

The persistence of these olfactory alterations in clinically improved patients may have different explanations. First, the persistence of olfactory impairments despite euthymia could be due to the repetition of depressive event and the chronicity of this disease. Thus, we assume that the patients’ olfactory and cognitive abilities after 6 weeks of antidepressant treatment were not completely restored compared to healthy volunteers. Indeed, many authors have already observed that biological and cognitive markers of major depression are not improving after antidepressant treatment. For instance, some authors [33] have shown that fluoxetine did not restore brain activity in mice. Besides, other authors have described the persistence of cognitive impairments in remitted patients after a MDE [34]. In our study, we used a selective serotonine reuptake inhibitor (SSRI, escitalopram) with only weak affinity for dopamine transporters. Because of the major implication of dopamine in depression [35] and in olfactory mechanisms [36]–[39], it is possible to show that this antidepressant treatment can’t normalize some cognitive impairments in clinically improved patients as olfactory ones.

In addition, depressed subjects performed weakly in identifying correctly the components in the binary iso-intense mixture, during the MDE and after clinical improvement. Our data also demonstrated that the depressed patients tended to perceive more the unpleasant compound compared to the controls (marginal difference, p = 0.06). This observation suggests that the loss of appetite frequently described during MDE could be partly explained by this modification in olfactory perception, which is expressed as an “olfactory negative alliesthesia”.

This is the first study to explore olfactory perception of complex odorant environment in clinically improved patients. In everyday life, subjects are confronted to complex odorant mixtures (e.g., food, beverages, perfumes, flowers, etc.). This experiment is of great interest because it reflects more the reality of one patient’s olfactory environment. This innovative approach paves the way for future studies aiming at investigating olfactory alterations in neuropsychiatric disorders.

The present study brings new evidence about olfactory impairments associated with MDE. Different olfactory impairments were tested as potential state or trait olfactory markers for MDE. Our results confirm the “olfactory anhedonia”, expressed by a decrease of hedonic score for high emotional odorant, as a potential state marker for MDE. Our prospective results revealed the persistence of an “olfactory anhedonia” for everyday life perceived odorants, an “olfactory negative alliesthesia” at a quantitative level (odor intensity evaluation) and a failure to identify two odorants with opposite valences in a binary iso-mixture, as potential trait markers for MDE. Moreover, this study underlined the importance of using complex odorant mixtures for a better understanding of the olfactory perception in mood disorders. Such a negative bias has already been described in previous studies investigating other types of stimuli in depression, e.g., a facial expression recognition bias in depression [40], [41]. Moreover, Mikhailova et al. (1996) [42] hypothesized a state deficit in emotion processing in depressed patients by evaluating the patients before treatment and after achieving remission.

Some limitations of this preliminary work must be considered. First of all, our observations need to be confirmed by further studies. Besides, it could be relevant to create standardized instruments using pure compounds with different hedonic valences (pleasant, unpleasant and neutral). It is important to understand the role of the hedonic valence of the olfactory compounds and the effect of specific odorants evoking strong memories and emotions. Moreover, to generalize our findings, we need to confirm them with a larger sample including several age ranges. Indeed, the average age of our participants is quite high (50 years) and it is known that olfactory capacities decrease with age [43]. Longitudinal studies are required to examine cognitive and olfactory differences in depressed subjects following remission from depression, in order to confirm potential state and trait markers for depression. Moreover, it would be necessary in further studies to include patients “at risk”, before the beginning of an acute MDE to see if some olfactory markers could constitute a risk factor of this disease. Besides, future studies could test olfactory performances in patients treated with another antidepressant treatment and other therapeutic methods in order to understand the possible differential influence of drugs and psychotherapies on the olfactory perception. At last, we can also hypothesize that our results could be partly due to the reduced interest during depression in their surroundings, reduced ability to concentrate on a task or their general negative mood; this aspect must be controlled in further studies.
